# 24. A case of necrotising autoimmune myopathy with recent statin use

**DOI:** 10.1093/rap/rky033.015

**Published:** 2018-09-20

**Authors:** Mahima Charan, Mithun Chakravorty, Thron Miah, Shahzad Akbar, Samir Al-Mitwally, Eva Palkonyai, Shaza Obaid

**Affiliations:** 1 Diabetes and Endocrinology, East, Midlands, Lincoln, UNITED KINGDOM; 2 Rheumatology, East Midlands, Lincoln, UNITED KINGDOM

**Introduction:** Inflammatory myopathies are a rare group of diseases that involve chronic muscle inflammation accompanied with muscle weakness. There are four main subtypes: polymyositis, dermatomyositis, inclusion body myositis, and necrotising autoimmune myopathy (NAM). In 2015, the incidence of inflammatory myopathy was 19 cases/million/year in the UK. NAM is a rarer subgroup which typically presents as a subacute, symmetrical proximal myopathy with significantly raised creatinine kinase (CK) levels. Weakness of neck flexion, dysphagia, dyspnoea and myalgia frequently occur without any ocular involvement. Characteristic histopathological findings on muscle biopsy are necrotic and regenerating fibres with minimal to no inflammation. Patients most at risk of developing NAM include those with statin exposure, connective tissue disease, malignancy and blood-borne viruses such as HIV and Hepatitis C. Statins are well-established medications in the treatment of cardiovascular and cerebrovascular disease, and taken by over seven million people in the UK. Statin-induced myopathy is a clinical spectrum of muscle related side-effects with an incidence of about 1.5-5.0% in randomised controlled trials and up to 25% in observation studies. The risk of developing statin-induced autoimmune inflammatory myositis is much rarer, with a prevalence of 1 in 100, 000. This case highlights this severe side-effect which necessitates potent immunosuppressive treatment, and further investigations and monitoring.

**Case description:** A 66 year old man presented to the acute medical ward with insidious onset of progressive muscle weakness and myalgias over three months. He had a background of type 2 diabetes mellitus, hypertension and ischaemic heart disease, and started treatment with atorvastatin 80 mg at night five months ago. This was stopped after three months in view of his symptoms, but his weakness continued to progress despite this. Eventually he became unable to mobilise from a seated position and had an environmental fall which prompted hospital admission. There was no significant injury or long lie on the ground. No other muscles were affected and there was no focal neurological symptoms. There was mild dyspnoea on exertion but without infective symptoms or symptoms of cardiac failure. He had not lost any weight and no symptoms to suggest underlying malignancy or connective tissue disease. There was no preceeding illness, recent travel abroad or illicit drug use. His regular medications included aspirin 75 mg daily, atenolol 50 mg daily, bendroflumethiazide 2.5 mg daily, doxazosin 2 mg three times daily, exenatide 10 mcg subcutaneously twice daily, gliclazide 80 mg twice daily, metformin 1 g twice daily and ramipril 10 mg daily. He had no known drug allergies. There was no significant family history. He had never smoked cigarettes and only drank alcohol occasionally. On examination he was alert, GCS 15/15, with a national early warning score (NEWS) of 0. Full neurological examination demonstrated 3/5 proximal weakness in upper limbs and 2/5 in lower limbs but preserved power distally. Reflexes, sensation and cranial nerves were normal. Physical examination of cardiovascular, respiratory and gastrointestinal symptoms was unremarkable. Initial blood tests showed normal full blood count, urea and electrolytes, estimated glomerular filtration rate above 90 ml/min and normal thyroid function tests. CK was more than 10, 000 U/L, AST 275 U/L, ALT 337 U/L, CRP 12 mg/L, ESR 18 mm/h. Chest x-ray, 12 lead electrocardiogram, NT-proB-type natriuretic peptide and non-invasive liver screen including viral and autoimmune hepatitis screen, and HIV test were normal. Electromyogram (EMG) was performed promptly and showed widespread active denervation changes associated with low amplitude spiky polyphasic motor units and early recruitment pattern, predominantly in the proximal limb muscles; consistent with inflammatory myopathy or myositis. neurology and rheumatology referrals were made by the medical team and subsequently CT chest, abdomen, pelvis was performed. This was unremarkable and tumour markers were also normal. Initial immunology tests antinuclear antibody (ANA) and extractible nuclear antigens (ENA) were negative and myositis-specific antibodies were requested and muscle biopsy arranged. At this stage, oral prednisolone 60 mg daily was commenced with gastric and bone protection, and close monitoring of capillary blood glucose. There was a significant improvement of symptoms within 48 hours as reported by the patient and objective assessment of proximal power improved to 3/5 in all four limbs. CK had reduced to 4, 129 by day nine of treatment, with mild improvement of liver function tests. Muscle biopsy showed necrotic and regenerative fibres, suggesting a necrotising inflammatory process. Myositis-specific antibodies were all negative except for HMGCR which is still pending. In view of the biopsy results, he is being considered for steroid-sparing agents early on whilst weaning steroids slowly. There is residual 4/5 proximal muscle weakness despite most recent CK improving to 1283 on day 27 of treatment. He has had regular allied health review during admission and is planned to undergo neuro-rehabilitation.

**Discussion:** An inflammatory myopathy was suspected early on in view of the very high CK and suggestive EMG findings. It was decided to start oral prednisolone prior to obtaining results of the myositis-specific antibodies and muscle biopsy as these would take several weeks and not alter initial management. The patient had a body weight above 80 kg and was therefore treated with a relatively higher dose of prednisolone. Further immunosuppressive therapy such as methotrexate is being considered once liver enzymes have improved further. There have been very few published reports of NAM. Nearly one third (34%) of cases are related to statins but more than 50% are idiopathic. Almost 10% are paraneoplastic with the commonest malignancy being gastrointestinal. Other cancers such as breast and lung cancer have also been associated with NAM. Only 4% have been associated with connective tissue disease with systemic lupus erythematosus (SLE) being the most common. NAM is associated with two myositis-specific antibodies in around two thirds of cases: signal recognition particle (anti-SRP) antibodies and hydroxy-3-methylglutaryl-coenzyme A reductase (anti-HMGCR) antibodies. Anti-SRP antibodies are not specific for NAM as they are also found in patients with systemic sclerosis and anti-synthetase syndrome. When present in NAM, it is associated with more systemic involvement e.g. cardiac and interstitial lung disease and refractory disease requiring more than one immune treatment. Anti-HMGCR antibodies have high sensitivity and specificity for NAM associated with statin use and patients in this subgroup usually respond well to treatment. However, when positive in statin-naive patients multiple treatments are usually required to control the disease. High dose corticosteroids are generally considered first line but usually require addition of immunosuppressives such as methotrexate, azathioprine, cyclophosphamide, mycophenolate mofetil and rituximab. Early use of intravenous immunoglobulin (IVIG) and plasmapheresis appears to have strength improvement and positive outcomes from several case reports.

**Key Learning Points:** NAM is a rare inflammatory myopathy which is most associated with statins and requires high dose immunosuppressive treatment in addition to stopping the statin. It is important to consider malignancy and connective tissue disease as other causes, and blood-borne viruses in high risk patients. Myositis-specific antibodies can guide treatment and prognosis in NAM e.g. anti-SRP antibody positive patients and anti-HMGCR antibody positivity in statin-naive patients. These patients often need multiple immunosuppressive treatments and may benefit from early IVIG or plasmapheresis. Liver function tests particularly transaminases are commonly abnormal in inflammatory myopathies without any underlying cause and usually normalise with treatment.

**Disclosure: M. Charan:** None. **M. Chakravorty:** None. **T. Miah:** None. **S. Akbar:** None. **S. Al-Mitwally:** None. **E. Palkonyai:** None. **S. Obaid:** None.

